# Editorial: Cancer immunotherapies for hematologic malignancies - NK/CIK/CAR-T perspective

**DOI:** 10.3389/fimmu.2024.1434259

**Published:** 2024-06-18

**Authors:** Amit Sharma, Antonio Rosato, Ingo G. H. Schmidt-Wolf

**Affiliations:** ^1^ Department of Integrated Oncology, Center for Integrated Oncology (CIO), University Hospital of Bonn, Bonn, Germany; ^2^ Department of Stereotactic and Functional Neurosurgery, University Hospital of Bonn, Bonn, Germany; ^3^ Department of Surgery, Oncology and Gastroenterology, University of Padova, Padua, Italy

**Keywords:** cancer immunotherapies, CIK cells, CAR-T cells, NK cells, cancer

Undeniably, immunotherapy has revolutionized the treatment of hematologic malignancies. Specially, NK/CIK/CAR-T cell therapies have shown tremendous success in the treatment of these clinically challenging malignancies (1–3). Besides, the efforts to integrate immune checkpoint blockades, novel inhibitors, and other immune/cancer cell therapies have contributed significantly to the unprecedented success of these aforementioned therapies across basic and translational immunotherapy research. However, a subset of patients continues to be unresponsive, thus emphasizing the need to further improve the therapeutic paradigm.

Therefore, it is of utmost importance to address ongoing and emerging strategies to advance NK/CIK/CAR-T immunotherapies in the preclinical and clinical settings. In the framework of this Research Topic, there have been five studies that have not only addressed the aforementioned concerns, but have also highlighted new perspectives.

Xia et al. used single cell RNA sequencing (scRNA-seq) to visualize the landscape of immune microenvironment in matched bone marrow and peripheral blood samples from three multiple myeloma (MM) patients undergoing anti-BCMA CAR-T therapy. The authors demonstrated tumor cell heterogeneity in different patients and reported a high-risk subpopulation that would resist CAR-T therapy. In addition, they found decreased T cell activity in relapsed patient and pointed out the importance of endogenous immunity in CAR-T therapy. Overall, the study provided new insights into the changes in endogenous immune cells, especially over a relatively long period of time after CAR-T therapy. Of interest, Wang et al. focused specifically on the therapeutic efficacy of interleukin 2 (IL-2) in cancer landscape. The authors described the effect of combining IL-2-loaded nanoparticles with CDK4/6 inhibitors (palbociclib), which resulted in superior tumor suppression without increased toxicity in mice. The study suggests that a new paradigm of combination therapy presented in their work could reshape cancer immunotherapy.

Noteworthy are also the case reports related to this Research Topic. Mu et al. focused on anaplastic large cell lymphoma (ALCL), one of the most common subtypes of T-cell lymphoma, especially refractory and relapsed (r/r) ALK-positive ALCL, for which there are no effective therapies. The authors reported the case of an ALK^+^ ALCL patient who had failed multiple chemotherapies and received an infusion of anti-CD5 CAR T cells. The study showed complete remission, sustained disease-free survival and manageable side effects. Thus, suggesting that anti-CD5 CAR T cells could be a new therapeutic approach for the treatment of advanced or relapsed ALK^+^ ALCL. Likewise, Zhang et al. presented the first successful case of combination therapy with autologous stem cell transplantation and CAR T cells for the treatment of relapsed and refractory CD20-negative transformed follicular lymphoma (tFL) with TP53 mutation and a massive mass. A critical condition of an adult patient initially diagnosed with follicular lymphoma that transformed into diffuse large B-cell lymphoma was comprehensively described. Moreover, the treatment success in the present report emphasizes the potential therapeutic strategy in tFL and provide rational for further investigation in a large population. Jiang et al. also described successful treatment with preventive donor-derived anti-CD7 CAR-T therapy in a case of refractory T lymphoblastic lymphoma after allogeneic hematopoietic stem cell transplantation (allo-HSCT). Notably, the authors reported complete remission and long-term disease-free survival with manageable side effects. Preventative CD7 CAR T-cell therapy after allo-HSCT has therefore been suggested to be an effective treatment for patients with refractory T-cell acute lymphoblastic leukemia/lymphoma (T-ALL/LBL).

Collectively, this Research Topic has brought forth diverse strategies and perspectives for the clinical application of cancer immunotherapeutic approaches. We are convinced that further in-depth research on NK/CIK/CAR-T approaches will lead to significant progress in the future, which will ultimately benefit a large number of people with cancer.

## Author contributions

AS: Writing – original draft, Writing – review & editing. AR: Writing – original draft, Writing – review & editing. IS: Writing – original draft, Writing – review & editing.
